# Supplementary material for: The Possible Impact of Zinc-Enriched Multivitamins on Treatment-Naïve Recurrent Aphthous Stomatitis Patients
Source: J Clin Med. 2025 Jan 5;14(1):260. doi: 10.3390/jcm14010260 (PMC11721099; doi:10.3390/jcm14010260)
Supplement: Supplementary file 1 [file jcm-14-00260-s001.zip › jcm-3395165-supplementary.pdf]

Table S1. Logistic regression analysis for the association of each variable with responders to treatment with ZnVita for RAS patients (n=134)

	Odds Ratio	95% Confidential Interval	p-value
Age at presentation, year	1.003	0.976-1.031	0.816
Duration of disease, year	0.998	0.955-1.043	0.930
Sex			
Male	0.584	0.252-1.352	0.209
Female*	1*		
Associated symptom			
Oral aphthae only*	1*		
Presence of BD-related symptoms	0.435	0.195-0.969	**0.042**†
NLR	0.759	0.393-1.464	0.411
MLR	12.842	0.023-6492.7	0.422
PLR	1.004	0.993-1.016	0.464
MPV/PC	0.025	$0.000\text{-}8.58*10^{17}$	0.872
ESR	0.995	0.969-1.021	0.687
CRP	0.994	0.959-1.030	0.729
HLA-B51 genotype*			
Positive	0.771	0.323-1.840	0.558
Negative*	1*		

*Reference category.

Significant differences are marked by † (p < 0 01).

Abbreviations; BD, Behçet’s disease, NLR, neutrophil-to-lymphocyte ratio; MLR, monocyte-to-lymphocyte ratio; PLR, platelet-to-lymphocyte ratio; MPV, mean platelet volume; PC, platelet count; ESR Erythrocyte sedimentation rate ; CRP, C-related protein.
